# Zinc Oxide Nanoparticles Catalyzed Condensation Reaction of Isocoumarins and 1,7-Heptadiamine in the Formation of Bis-Isoquinolinones

**DOI:** 10.1100/2012/619080

**Published:** 2012-03-12

**Authors:** Varadhan Krishnakumar, Kesarla Mohan Kumar, Badal Kumar Mandal, Fazlur-Rahman Nawaz Khan

**Affiliations:** ^1^Organic Chemistry Division, School of Advanced Sciences, VIT University, Tamil Nadu, Vellore 632 014, India; ^2^Trace Elements Speciation Research Laboratory, Environmental and Analytical Chemistry Division, School of Advanced Sciences, VIT University, Tamil Nadu, Vellore 632 014, India

## Abstract

The diversified bis-isoquinolinones were obtained in two steps, utilizing homophthalic acid and various acid chlorides providing 3-substituted isocoumarins in the first step which on further condensation with 1,7-heptadiamine involving C–N bond formation from the lactone in the presence of 10 mol% zinc oxide nanoparticles (ZnO NPs) (<150 nm) afforded the desired bis-isoquinolinones in high yield and purity. The synthesized compounds were then characterized using FTIR, ^1^H NMR, ^13^C NMR, and HRMS techniques.

## 1. Introduction

The isoquinolinones form the core structures of plant alkaloids [1] and biologically important compounds [2–5]. In recent decades, isoquinolinones have been used as a precursor of phenylisoquinoline-iridium complex, a high-efficiency red phosphorescent dopant, in organic light-emitting diodes (OLEDs) [6, 7]. There are many reports for their synthesis including cyclization of 2-chlorobenzonitriles and keto esters [8], condensation of 2-(bromomethyl)benzonitriles [9], 2-methylbenzonitrile and 2-methylbenzamide [10], 2-halobenzaldimines and alkynes [11], denitrogenative addition of benzotriazinones and alkynes [12], double metalation of arylbenzamides [13, 14], *N*-pyridinylphthalimide and alkynes [15], and the metal-mediated synthesis of isoquinolinones has also been successfully emerged [16–18]. In our group, we have already reported the various 3-substituted isoquinolinones from isocoumarins [19, 20]. Most of the above methods have limitations such as long reaction time, low yield, tedious workup procedure, and use of expensive, toxic reagents or catalysts. Indeed, methods involving C–O bond cleavage with subsequent C–N bond formation are emerging as attractive alternatives for isoquinolinones and that nanosized particles are considered to be attractive as catalysts for their greater reactivity, due to high surface area, recovered easily from the reaction mixture and reutilized for further reaction, therefore the method being more economical [21]. The development of efficient methods for their synthesis is of great importance, and we describe an efficient ZnO NPs mediated reaction for the production of bis-isoquinolinones.

## 2. Experimental

### 2.1. Materials and Methods

**Scheme 1:**
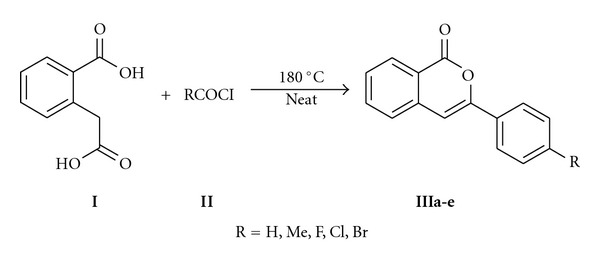
Synthesis of 3-substituted isocoumarins **IIIa-e**.

The chemicals and reagents used were purchased from Sigma Aldrich (India) and were used as purchased. Melting points were measured using open capillary tubes and are corrected with reference to benzoic acid. IR spectra (in KBr pellets) were recorded on Nucon Infrared spectrophotometer (India). ^1^H NMR (400 MHz and 500 MHz) and ^13^C NMR (100 MHz and 125 MHz) spectra were recorded on a Bruker 400 MHz and 500 MHz spectrometer in CDCl_3_ using TMS as a standard. High-resolution mass spectra (HRMS) were obtained using JEOL GC MATE II HRMS (EI) mass spectrometry. The isocoumarins (**III**) required for the study were obtained following our earlier report [22] from the homophthalic acid (**I**) and corresponding acid chlorides (**II**) (Scheme 1).

### 2.2. Synthesis of ZnO Nanoparticles

The ZnO NPs required for the present investigation were obtained from hydrolysis of zinc acetate using sodium hydroxide as per Hu et al.'s methodology [23] with minor modification as follows. Zinc acetate dihydrate 2.2 g was dissolved in 300 mL of milli-Q water. NaOH 8 g was dissolved in 50 mL of milli-Q water. Then later solution was added dropwise into zinc acetate solution using a peristaltic pump (Revotek, India) and stirred vigorously using a magnetic stirrer for half an hour. The resulting turbid solution was placed on a temperature-controlled water bath at 90°C for one hour, and thus formed white powder was separated, washed with milli-Q water (25 mL × 2) and with ethanol (25 mL × 2), and then subjected to calcination at 300°C for 4 h.

### 2.3. Characterization of ZnO Nanoparticles

The ZnO NPs prepared were characterized using powder X-ray diffraction (XRD), transmission electron microscope (TEM), and energy dispersive X-ray spectroscopy (EDX). The XRD was performed using Bruker D8 Advance diffractometer with Cu K*α* radiation (*λ* = 1.54 Å). TEM images were taken (Philips TEM, Netherlands) by dropping a drop of sample dispersion (1 mL samples added to 5 mL of propanol and sonicated for few minutes) on a carbon-coated copper grid and dried in vacuum. The instrument was operated with an acceleration voltage of 100 Kv. Simultaneously EDX spectrum was recorded at selected areas on the solid surface to obtain the information on surface atomic distribution. Average particle size and size distribution were determined according to CPS Disc Centrifuge particle size analyzer.

### 2.4. General Procedure for the Synthesis of Bis-Isoquinolinone Derivatives (**Va-e**)

#### 2.4.1. Method A

The condensation of 3-substituted isocoumarins (**IIIa-e**) and 1,7-heptadiamine (**IV**) was carried out in 100 mL round-bottom flask fitted with Dean-Stark trap for water removal and a reflux condenser. The reaction was performed by refluxing 1 mmol of 3-substituted isocoumarins (**III**) 0.5 mmol of 1,7-heptadiamine (**IV**) in the presence of catalytic amount of paratoluene sulphonic acid (pTSA) and 20 mL of dry toluene as a solvent. The progress of the reaction was monitored by thin layer chromatography. After completion of the reaction, the solvent was removed under vacuum, the crude product (**V**) was dissolved in 20 mL of ethyl acetate and washed with water, then the organic layer was passed through anhydrous sodium sulphate, concentrated the solvent, and then it was purified by silica gel column chromatography using ethyl acetate and petroleum ether (15 : 85) mixture as an eluant.

#### 2.4.2. Method B

A mixture of 3-substituted isocoumarins (**IIIa-e**) and 1,7-heptadiamine (**IV**) (1 : 0.5 ratio) in dry toluene in the presence of 10 mol% ZnO NPs was heated under reflux condition. The progress of the reaction was monitored by thin layer chromatography. After completion of the reaction, the catalyst was separated by filtration. The solvent was removed under vacuum, and then the crude product (**V**) was purified by silica gel column chromatography using ethyl acetate and petroleum ether (15 : 85) mixture as an eluant. The purified products (**Va-e**) were characterized by FTIR, ^1^H NMR, ^13^C NMR, and HRMS techniques. The spectral data of compounds (**Va-e**) are given in Table 4.

## 3. Results and Discussion

**Figure 1:**
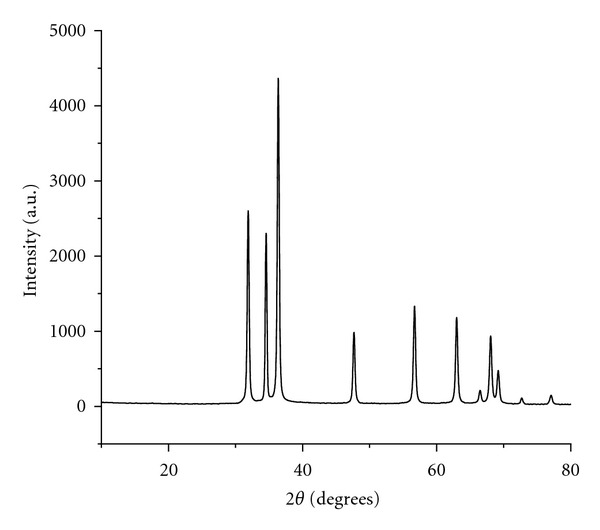
XRD pattern of ZnO NPs.

**Figure 2:**
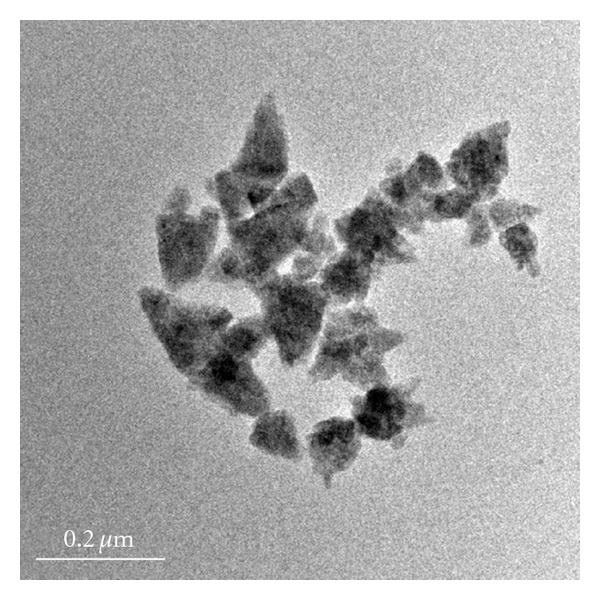
TEM image of ZnO NPs.

**Figure 3:**
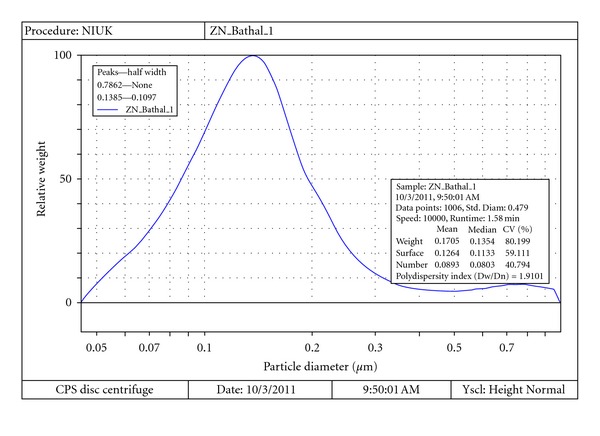
Particle size analyzer.

**Figure 4:**
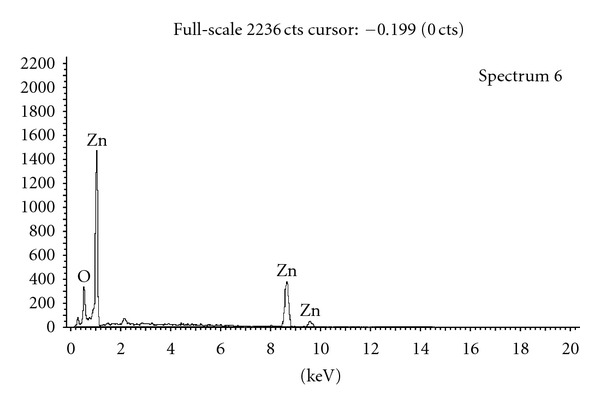
EDX spectrum of ZnO NPs.

Presently the ZnO NPs required for the investigation were obtained in high purity and yield from the corresponding zinc acetate solution. The XRD pattern of ZnO NPs suggested discrete diffraction peaks at 31.90, 34.40, 36.37, 47.67, 56.71, 62.98, 66.51, 68.05, and 69.21 which can be assigned to (110), (002), (101), (102), (110), (103), (200), (112), and (120) of hexagonal ZnO NPs (Figure 1) and that the lattice constants were in agreement with the standard card (JCPDS no. 00-0361451). The TEM image of ZnO NPs revealed the average particle size of less than 150 nm (Figure 2) and further supported by particle size analysis (Figure 3). The EDX spectrum of the nanosample also confirmed the presence of Zn and O atoms (Figure 4).

**Scheme 2:**
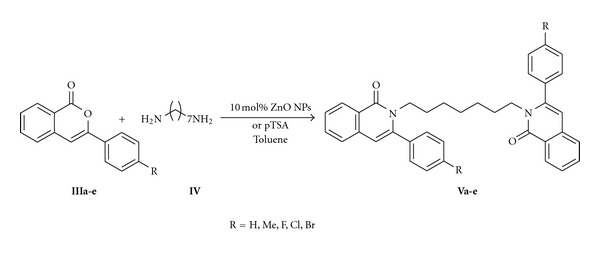
Synthesis substituted bis-isoquinolinones **Va-e**.

The 3-substituted isocoumarins required for the investigation were obtained following our earlier report [22]. And preliminarily, the bis-isoquinolinones **V **were obtained from the isocoumarins **III** (1 mmol) and 1,7-heptadiamine **IV** (0.5 mmol) in the presence of catalytic amount of pTSA and dry toluene using Dean-stark apparatus (Scheme 2; Table 1). However, the reaction required longer time of 24–32 h for completion and the purity of products was not impressive. The formed products were purified and characterized by various spectral techniques such as FTIR, ^1^H NMR, ^13^C NMR, and HRMS techniques.

In the search of reducing the reaction time and to improve the yield and purity of the products, it was evidenced that indium trichloride [24] was an effective catalyst; however, due to their cost and toxicity [25, 26], we envisioned the application of an inexpensive and environmentally benign catalysts. The optimization of the reaction was carried out by varying the catalyst, solvent, reactant substituents.

Initially, the ZnO (bulk) was used as a catalyst for the reaction. In our attempt, in the presence of 5 to 25 mol % ZnO (bulk) catalyst, the reaction continued until 36 h without formation of the desired product, **V**. Then, we investigated the presently synthesized ZnO NPs catalyst in the presence of dry toluene, and it was noticed that ZnO NPs (10 mol%) mediation improved the reaction yield along with reduced time of 10–12 h (Table 1 entry 2).

The optimization of the catalyst was done using the reaction between 3-(4-fluorophenyl)-1*H*-isochromen-1-one (**IIIc**) and 1,7-heptadiamine (**IV**) in the presence of ZnO NPs to afford the product **Vc** in high yield (Table 1). Further, it was noticed that the effect of solvent plays a role towards reaction time and that in benzene solvent the yield and purity was increased with reduced reaction time period (Table 2).

The effect of substituents on the reaction was also investigated, and it was observed that the rate of the reaction (Table 2) was increased in the presence of electron donating substituents (entry b) compared to electron withdrawing substituents (entries c, d, and e); however, in the case of nitrosubstitution, there is no progression in the reaction, even after the reaction continued up to 36 hours (entry f).

**Figure 5:**
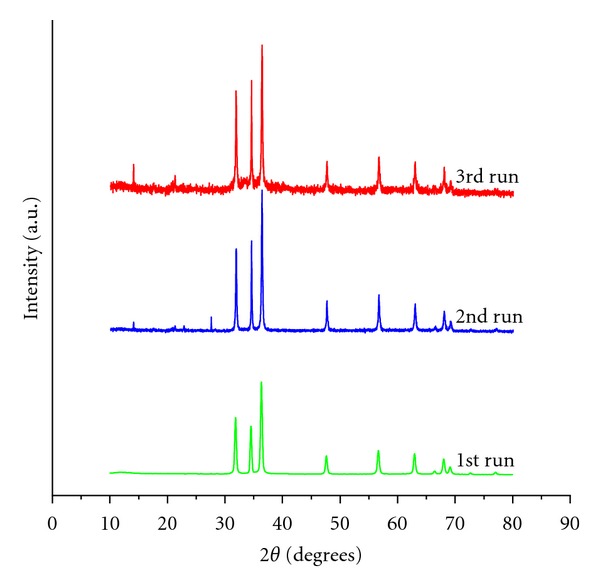
XRD spectrum of reutilized ZnO NPs.

The reusability of the catalyst was investigated by considering the reaction between **IIIc** and **IV** to afford **Vc**. The catalyst was recovered from the reaction mixture easily by filtration after first run. Then, it was washed with distilled water and ethyl alcohol three times and dried at hot air oven for 1 h. The recovered catalyst was unaltered (Figure 5) and used thrice to obtain the desired product without considerable loss of yield 89, 88, and 87%, respectively. It is noteworthy that the yield of the product in the first, second, and third runs was almost the same as that in the fresh catalyst run (Table 1). 

A comparison has been made between the pTSA and ZnO NPs as represented in Table 3. The physical data of bis-isoquinolinones are given in Table 4.

**Figure 6:**
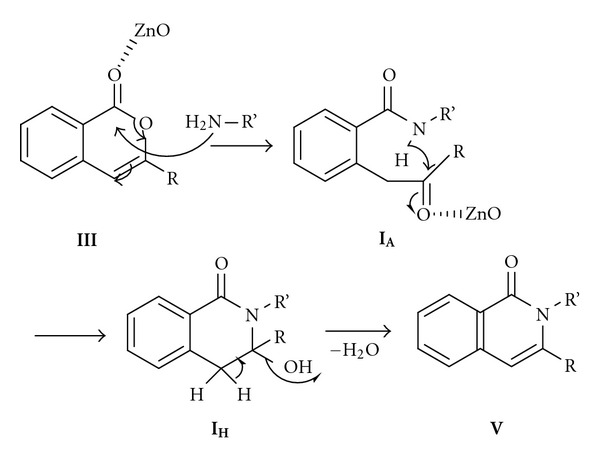
Plausible mechanism for the amination of isocoumarins.

The plausible mechanism involving the role of ZnO NPs in the condensation of isocoumarins and diamine to afford the corresponding isoquinolinones are given as in Figure 6. The mechanism gets initiated through the activation of the carbonyl group by the ZnO NPs with the subsequent nucleophilic attack of the amines to form the corresponding **I_A_** amide derivatives which then cyclized again with the mediation of ZnO NPs affording the corresponding hydroxyl derivative **I_H_** which then facilitates the elimination of water to afford the bis-isoquinolinones **V**, the catalyst regenerated then activate further isocoumarins and the cycle is continued.

### 3.1. HRMS, FTIR, and NMR Spectra

The HRMS of bis-isoquinolinones **V** showed a molecular ion peak M+ in the positive mode. The molecular ion peak of **Va** was observed at m/z = 537.8527, similarly, that of others (**Vb-e**) were observed at m/z 565.7487, 574.5888, 606.8743, and 696.3511, respectively.

In FTIR spectrum, the bis-isoquinolinones (**Va-e**) inferred the carbonyl frequency at around 1646-1647 cm^−1^. The bis-isoquinolinones **V** are symmetric with respect to the middle carbon of the heptyl group, and hence there are four magnetically equivalent carbons which appeared in the aliphatic region; the corresponding protons resonated at lower chemical shifts. Similarly, the two CH protons of the isoquinolinone rings appeared as singlet in ^1^H NMR and the aromatic ring at its 3-position and the other protons of the isoquinolinone ring appeared at higher chemical shifts. The ^13^C NMR peaks appeared between *δ* 162.70–162.85 also indicated the symmetrical carbonyl groups of isoquinolinone rings.

## 4. Conclusion

In short, a green, inexpensive, and efficient method for the synthesis of bis-isoquinolinone using ZnO NPs as a reusable catalyst was established. This new strategy has the several advantages including mild conditions, high yield, efficient, and environmentally benign approach which overcomes the drawbacks of the earlier condensation methods.

## Figures and Tables

**Table 1:** Optimization of the catalyst.

S. no.	Mol %		
		ZnO (**Vc**, yield %)	
		Bulk	Nano
1	5	trace	55
2	10	trace	89^a^, 89^b^, 88^c^, 87^d^
3	15	trace	90
4	20	trace^e^	90

^a^fresh, ^b^1st run, ^c^2nd run, ^d^3rd run, ^e^ZnO (bulk) tried up to 30 mol%.

**Table 2:** Effect of solvent on 10 mol% ZnO NPs mediated amination of isocoumarins.

Compound	R	Time in hrs		
			Yield %	
			Toluene	Benzene
**Va**	H	12	70	72
**Vb**	CH_3_	12	75	76
**Vc**	F	10	89	90
**Vd**	Cl	10	84	85
**Ve**	Br	10	80	83
**Vf**	NO_2_	36	—	—

**Table 3:** Comparison of pTSA and ZnO NPs.

Compound	R	Time in hrs	Yield %		
		pTSA	Nano ZnO	pTSA	Nano ZnO
**Va**	H	32	12	60	70
**Vb**	CH_3_	32	12	62	75
**Vc**	F	24	10	70	89
**Vd**	Cl	26	10	65	84
**Ve**	Br	26	10	69	80
**Vf**	NO_2_	36	36	—	—

**Table 4:** Physical data.

**Va**	White solid, mp 121–123°C, IR cm^−1^ 1647 (C=O), 1336 (C–N), ^1^H NMR (400 MHz, CDCl_3_) *δ* 0.96 (m, 6H), 1.46 (m, 4H), 3.86 (t, 4H, *J* = 8.0 Hz, N-*CH_2_*), 6.38 (s, 2H), 7.37–7.63 (m, 16H), 8.44 (d, 2H, *J *= 4.0 Hz) ppm. ^13^C NMR (100 MHz, CDCl_3_) *δ* 26.55, 28.32, 28.61, 45.55 (symmetrical aliphatic carbons), 107.84, 125.34, 125.83, 126.63, 128.01, 128.81, 128.98, 129.14, 132.32, 136.21, 136.39, 143.75, 162.85 (symmetrical carbonyl carbons) ppm, HRMS [EI, M+] calcd for C_37_H_34_N_2_O_2_ m/z 538.2620, found 537.8527.

**Vb**	Off white solid, mp 110–112°C, IR cm^−1^ 1647 (C=O), 1339 (C–N), ^1^H NMR (400 MHz, CDCl_3_) *δ* 0.88 (m, 6H), 1.25 (s, 6H), 1.45 (m, 4H), 3.85 (t, 4H, *J* = 4.0 Hz, N-*CH_2_*), 6.35 (s, 2H), 7.26–7.47 (m, 14H), 8.41 (d, 2H, *J* = 8.0 Hz) ppm; ^13^C NMR (100 MHz, CDCl_3_) *δ* 26.62, 28.40, 28.70, 29.90, 45.58 (symmetrical aliphatic carbons), 107.80, 125.49, 125.83, 126.65, 128.21, 128.57, 129.04, 129.20, 132.31, 135.13, 136.45, 143.79, 162.77 (symmetrical carbonyl carbons) ppm, HRMS [EI, M+] calcd for C_39_H_38_N_2_O_2_ m/z 566.2933, found 565.7487.

**Vc**	White solid, mp 117–119°C, IR cm^−1^ 1646 (C=O), 1336 (C–N), 694 (C–F), ^1^H NMR (500 MHz, CDCl_3_) *δ* 1.02 (m, 6H), 1.48 (m, 4H),), 3.88 (t, 4H, *J* = 7.5 Hz, N-*CH_2_*), 6.37 (s, 2H), 7.28–7.66 (m, 14H), 8.44 (d, 2H, *J* = 8.0 Hz) ppm. ^13^C NMR (125 MHz, CDCl_3_) *δ* 26.41, 28.39, 28.62, 45.45 (symmetrical aliphatic carbons), 107.95, 123.21, 125.35, 125.77, 126.80, 127.99, 130.68, 131.69, 132.33, 135.13, 136.07, 142.37, 162.67 (symmetrical carbonyl carbons) ppm, HRMS [EI, M+] calcd for C_37_H_32_F_2_N_2_O_2_ m/z 574.659, found 574.5888.

**Vd**	Pale yellow solid, mp 135–138°C, IR cm^−1^ 1646 (C=O), 1337 (C–N), 687 (C–Cl), ^1^H NMR (400 MHz, CDCl_3_) *δ* 1.03 (m, 6H), 1.45 (m, 4H), 3.85 (t, 4H, *J *= 7.6 Hz, N-*CH_2_*), 6.35 (s, 2H), 7.26–7.64 (m, 14H), 8.41 (d, 2H, *J* = 0.4 Hz) ppm. ^13^C NMR (100 MHz, CDCl_3_) *δ* 26.41, 28.37, 28.60, 45.55 (symmetrical aliphatic carbons), 108.06, 125.29, 125.77, 126.80, 127.96, 128.72, 130.40, 132.34, 134.61, 135.03, 136.07, 142.31, 162.70 (symmetrical carbonyl carbons) ppm, HRMS [EI, M+] calcd for C_37_H_32_Cl_2_N_2_O_2_ m/z 606.1841, found 606.8743.

**Ve**	White solid, mp 125–127°C, IR cm^−1^ 1677 (C=O), 1321 (C–N), 696 (C–Br), ^1^H NMR (500 MHz, CDCl_3_) *δ* 1.02 (m, 6H), 1.48 (m, 4H), 3.88 (t, 4H, *J* = 7.5 Hz, N-*CH_2_*), 6.38 (s, 2H), 7.28–7.66 (m, 14H), 8.44 (d, 2H, *J* = 8.0 Hz) ppm. ^13^C NMR (125 MHz, CDCl_3_) *δ* 26.41, 28.38, 28.62, 45.46 (symmetrical aliphatic carbons), 107.98, 123.21, 125.33, 125.78, 126.81, 127.99, 130.68, 131.69, 132.34, 135.12, 136.07, 142.36, 162.68 (symmetrical carbonyl carbons) ppm, HRMS [EI, M+] calcd for C_37_H_32_Br_2_N_2_O_2_ m/z 696.4702, found 696.3511.
